# Association between primary care coverage and breast cancer mortality in Brazil

**DOI:** 10.1371/journal.pone.0200125

**Published:** 2018-08-02

**Authors:** Francisco Winter dos Santos Figueiredo, Tábata Cristina do Carmo Almeida, Jean Henri Maselli Schoueri, Caio Luisi, Fernando Adami

**Affiliations:** Epidemiology and Data Analysis Laboratory, Faculdade de Medicina do ABC, Santo André, Brasil; University of Kentucky, UNITED STATES

## Abstract

Breast cancer early detection is the major strategy for mortality rates reduction. In Brazil, Primary Health Care is an important strategy for public health promotion. To analyse the association between breast cancer mortality and primary health care indicators in Brazilian municipalities, data on breast cancer mortality and primary healthcare coverage of the 5,700 Brazilian municipalities were collected from the Department of Informatics of the Brazilian Unified Health System. We collected data on the deaths of women living in Brazil in 2010 with breast cancer. Breast cancer mortality was calculated by 100,000 women and age-standardised from the World Health Organisation population. We studied the coverage of primary health care, family health team and community health agents. We found that increase of both primary care indexes was related to increasing of the breast cancer mortality. Additionally, improving the scholarly and reducing the income inequality was related to reducing the breast cancer mortality. Strategies to improve the quality of primary care, reduce the income inequality and improve elementary scholarly should be taken into account in the development of public policies in the Brazilian municipalities to reduce breast cancer in Brazilian municipalities.

## Introduction

Overall, one-third of all breast cancer cases worldwide could have been cured if they had been diagnosed early. This is because tumours in advanced stages show greater resistance to treatment, thus increasing the risk of dying from breast cancer [1].

The main international strategies to reduce breast cancer mortality rates are the reduction of inequalities in health services and the early diagnosis of cases[2]. For this to occur, there is a need to develop policies to bring the health service closer to the population, increase the number of tools available for early diagnosis (e.g. number of mammographs) and improve the coverage of mammographic screening in the target population[3].

Early diagnosis of breast cancer is determined by the triad: i) knowledge of the population regarding signs and symptoms; ii) professional qualification for diagnosis; and iii) the capacity of the health system to offer services to confirm the diagnosis and support the diagnosed cases[4].

In Brazil, where the breast cancer mortality was approximately 16 deaths per 100,000 inhabitants in 2014[5], this triad for early diagnosis may be related to the service provided in primary care. Some public policies carried out in primary care may be effective for the early diagnosis of breast cancer, even indirectly. In addition, regional differences in relation to socioeconomic inequities, ethnic, territorial extension and access to health services are related to differences in breast cancer rates among different geographical units [6, 7].

Among them, we highlight Primary Health Care (PHC), which carries out health promotion actions. PHC is made up of family health strategies, which are units including Family Health Team (FHTs) made up of health professionals and Community Health Agents (CHAs) who are those individuals in the community who are responsible for creating a closer link between the general population and health services.

The PHC is responsible for low and medium complexity health care for the population [8]. The FHS team is responsible for implementing health promotion actions in specific geographic areas and serves approximately 53% of the Brazilian population in 2011[9]. In addition, CHAs reached approximately 124 million people in Brazil, performing actions to identify health problem, need to health education and directing these cases to the FHS team [10].

These strategy are responsible for covering population areas and awareness of the signs and symptoms of diseases and facilitating access to health services, such as referral for mammograms to screen women in the target age range (50–69 years of age)[11].

However, in spite of all of the efforts of the Brazilian public service for the early detection of breast cancer (up to stage II), late diagnosis has seems to grow, varying from 45% of cases diagnosed in stage III or higher in 2005 to approximately 53% of cases in 2015 [12, 13]. Similarly, breast cancer mortality rates also increased during the first decade of the 21^st^ century[14, 15].

Thus, this study analysed the correlation between indicators of primary health care coverage (PHC coverage, FHS coverage, and HCAs coverage) with age-standardised breast cancer mortality in Brazilian municipalities. Our hypothesis is that in municipalities where there is greater coverage of primary care, there are lower rates of breast cancer mortality.

## Materials and methods

### Study design

An ecological study carried out in 2018 using data from 2010. Data were obtained from the Department of Informatics of the Unified Health System (DATASUS). The units of analysis were the 5,700 Brazilian municipalities.

### Database

#### Mortality Information Systems (SIM)

The SIM was the system used to identify the number of deaths in women. The SIM has been used in Brazil as a source of information on mortality since 1975, and provides data on the deaths, allowing the management of health services. It is an intermediate system, according to the World Health Organisation, where the quality and coverage of the system determine the reliability of the data[16, 17].

Coverage is influenced by the number of deaths that do not occur within the hospital setting and therefore may not be reported. This occurs mainly in less developed regions and of greater territorial extension such as the North and Northeast regions. In Brazil, data coverage is approximately 96%, ranging from 94% in 2009 to 96% in 2013[18, 19].

The quality of the system is measured by the number of deaths reported with ill-defined causes or with ignored/blank fields [18]. According to Adami et al. [20] the proportion of deaths due to ill-defined causes in relation to total deaths ranged from 7.4% in 2008 to 6.4% in 2012, demonstrating a good quality of the system because it reports a low proportion of cases with ill-defined causes.

#### Health Information System for Primary Care (SIAB)

The data for the measurement of the coverage of primary care indicators were obtained from the SIAB through the coverage history of the Family Health Coverage History available on the website of the Primary Care Department of SIAB (dab.saude.gov.br). SIAB provides data on Brazilian political strategies for primary care, through information on family health strategy, oral health strategy and community health agents [21].

### Studied variables

#### Mortality from breast cancer in women

Deaths from breast cancer were obtained from the SIM for each municipality by place of residence and age groups, in all females. For the classification of breast cancer, the code referring to malignant neoplasms of the breast was used (Code C50), according to the International Classification of Diseases (ICD-10) [22].

The resident population in Brazil was obtained from the 2010 Census, made available by the Brazilian Institute of Geography and Statistics (IBGE) by the Demographic and Socioeconomic Information Module of the Health Information System (TABNET/DATASUS).

The crude mortality rate was calculated using data for the number of deaths in the female resident population, per 100,000 women. The crude rate was age-standardised by the direct method based on the age of the world standard population of the World Health Organisation (WHO) [23].

#### Primary health care indexes

The average annual coverage of the Primary Health Care (*Atenção Básica* Coverage), the Family Health Team (FHT) coverage and the annual coverage of the Community Health Agents (CHA) were also extracted from the SIAB for each municipality[21]. The data were collected by each month in the 2010 year, and a mean between months was estimated to assess the annual coverage of PHC, FHT and CHA, respectively. The indexes of the Table 1 were calculated by the following calculate methods [21].

**Table 1:** Indexes, description and calculate method of the primary health care.

Indexes	Description	Calculate method
PHC Coverage	Populational coverage by the Basic attention composed by Family Health Strategy (family health teams and traditional basic attention teams).	$\frac{Number\;of\;Family\;Health\;Strategy*\;x\;3.450}{IBGE\;Population}$
FHT coverage	The proportion of the population covered by Family Health Team. This is an indicator of the accessibility to public health service.	$\frac{Number\;of\;Family\;Health\;teams\;x\;3.450}{IBGE\;Population}$
CHA coverage	The proportion of the population covered by Community Health Agents, used as a longitudinal indicator of primary attention.	$\frac{Number\;of\;Community\;Health\;Agents\;x\;450}{IBGE\;Population}$

#### Confounders

Some socioeconomic and inequity variables were collected to estimates adjustment. The sources used were Department of Informatics of the Unified Health System (DATASUS) and United Nations Development Programme (UNDP). The variables and sources were described in Table 2 below.

**Table 2:** Socioeconomic and inequity variables used for the adjustment.

Variables	Source
GINI index	UNDP
Human Development Index income	UNDP
Human Development Index longevity	UNDP
Human Development Index education	UNDP
Per capita income	UNDP
Poverty vulnerability (%)	UNDP
Proportion of people with 25 years-old having completed elementary education (%)	UNDP
Proportion of women with 10–17 years-old and with child (%)	UNDP
Hours of doctors in primary care	DATASUS
Hours of nurses in primary care	DATASUS

UNDP: United Nations Development Programme

DATASUS: Department of Informatics of the Unified Health System

### Data analysis

Descriptive statistics were used to present age-standardized breast cancer mortality and primary care indicators (PHC coverage, FHTs coverage and CHA coverage). The stepwise backward method was used to select the better model after inclusion or exclusion of the adjustment variables. The inclusion and exclusion criteria for the final models were p<0.20 and p<0.05, respectively. The better models were choice based on the explanatory capacity of the model (adjusted r^2^). The confidence interval was 5%. Stata 11.0 ^®^ (Stata Corp., College Station, EUA) was used.

## Results and discussion

Only in 2,305 Brazilian municipalities occurs deaths from breast cancer in 2010. The average of the age-standardized mortality for breast cancer was 14.5, ranged from 14.1 to 15.0 deaths per 100,000 women. Regarding the primary care indexes, the PHC coverage was 80.6% (varying from 79.7 to 81.6%), the FHC 71.7% (varying from 70.4 to 73.0%) and the CHA coverage was 80.7% (varying from 79.5 to 81.9%) (Table 3).

**Table 3:** Age-standardized mortality for breast cancer (per 100,000 women) and primary health care coverage in Brazilian municipalities in 2010.

Variables	Brazilian municipalities
	Mean (95% CI)
Age-standardized breast cancer mortality ^a^	14.5 (14.1; 15.0)
Coverage indexes	
Primary Health Care Coverage	80.6 (79.6; 81.6)
Family Health Teams Coverage	71.7 (70.4; 73.0)
Community Health Agents coverage	80.7 (79.5; 81.9)

95% CI: 95% confidence interval

^a^ age-standardized per 100,000 women by the direct method based on the age of them world standard population of the World Health Organization (WHO) [23].

In Table 4, we present the association between age-standardized breast cancer mortality and Primary Health Care (PHC) coverage. We observed that increase of the PHC coverage is related to 0.12 (varying from 0.11 to 0.15; p<0.001) deaths from breast cancer (per 100,000 women), an increase of the proportion of people with years-old having completed elementary education and Gini index were related to decreasing of mortality (-0.04, 95% CI -0.08; -0.002; p = 0.049 and -10.9, 95% CI -18.2; -3.5; p = 0.004, respectively).

**Table 4:** Association between age-standardized mortality for breast cancer (per 100,000 women) and primary health care coverage in Brazilian municipalities in 2010.

Variables	Breast cancer mortality ^a^	
	β (95% CI)	p*
Primary Health Care Coverage	0.12 (0.11; 0.15)	<0.001
Proportion of people with 25 years-old having completed elementary education	-0.04 (-0.08; -0.002)	0.049
Gini index	-10.9 (-18.2; -3.5)	0.004

*Linear regression adjusted by confounder variables

95% CI: 95% confidence interval

^a^ age-standardized per 100,000 women by the direct method based on the age of them world standard population of the World Health Organization (WHO) [23].

In Table 5, we present the association between age-standardized breast cancer mortality and the Family Health Teams coverage. In this aspect, the increase of the FHC coverage is related to increase of the breast cancer mortality in 0.09 (95% CI 0.08, 0.11; p<0.001) and, the proportion of people with years-old having completed elementary education was related to decreasing of breast cancer mortality in -0.10 (95% CI -0.19; -0.01; p = 0.03).

**Table 5:** Association between age-standardized mortality for breast cancer (per 100,000 women) and family health teams coverage in Brazilian municipalities in 2010.

Variables	Breast cancer mortality ^a^	
	β (95% CI)	p*
Family health Teams Coverage	0.09 (0.08; 0.11)	<0.001
Proportion of women with 10–17 years-old and with child	0.19 (-0.09; 0.57)	0.19
Proportion of people with 25 years-old having completed elementary education	-0.10 (-0.19; -0.01)	0.03
Human Development Index education	9.9 (-2.1; 21.9)	0.11
Gini index	-8.6 (-17.2; 0.03)	0.05

*Linear regression adjusted by confounder variables

95% CI: 95% confidence interval

^a^ age-standardized per 100,000 women by the direct method based on the age of them world standard population of the World Health Organization (WHO) [23].

In Table 6, the role of the Community Health Agents coverage to breast cancer mortality is presented. The increase of the CHA coverage is related to 0.06 (95% CI 0.04, 0.07; p<0.001) and the increase of the proportion of people with years-old having completed elementary education and Gini were related to the decreasing of the breast mortality (-0.12, 95% CI -0.21, -0.02; p = 0.01 and -10.0, 95% CI -18.9, -1.20; p = 0.03, respectively).

**Table 6:** Association between age-standardized mortality for breast cancer (per 100,000 women) and community health agents coverage in Brazilian municipalities in 2010.

Variables	Breast cancer mortality ^a^	
	β (95% CI)	p*
Community Health Agents coverage	0.06 (0.04; 0.07)	<0.001
Proportion of women with 10–17 years-old and with child (%)	0.24 (-0.04; 0.53)	0.10
Proportion of people with 25 years-old having completed elementary education (%)	-0.12 (-0.21; -0.02)	0.01
Human Development Index education	11.1 (-1.2; 23.3)	0.08
Gini index	-10.0 (-18.8; -1.20)	0.03

*Linear regression adjusted by confounder variables

95% CI: 95% confidence interval

^a^ age-standardized per 100,000 women by the direct method based on the age of them world standard population of the World Health Organization (WHO) [23].

When analysing the association among Primary Health Care (PHC), Family Health Team (FHT) and Community Health Agents (CHA) coverage, with age-standardised breast cancer mortality in the municipalities of Brazil, we found that: i) All primary care indexes were associated to of age-standardized breast cancer mortality; ii) the increase of the proportion of people with years-old having completed elementary education is related to decreasing of the mortality and; iii) Gini index was related to lower breast cancer mortality for Brazilian municipalities.

Several studies have analysed the association between indicators of primary care with outcomes related to the health of the Brazilian population [9, 24–26], and the impact of the breast cancer in Brazilian public policies[5], but none have analysed the breast cancer mortality as an outcome. To the best of our knowledge, this is the first study to analyse the association between primary care indexes in several aspects with breast cancer mortality in Brazilian municipalities.

We understand that there are some limitations to this study. The fact that it was carried out with data for one year, and being cross-sectional and data quality may have caused an information bias. The cross-section would illustrate a reality arising from this growth, but a longitudinal analysis may allow the space-time association to be estimated, as the increase in primary care coverage may have a modifying effect on the rates of mortality from breast cancer. On the other hand, it demonstrates a scenario where it takes time for the effects of primary health care coverage on population health to occur. This is because the increase in coverage of primary care indicators in Brazil is gradual.

In general, Primary Care becomes important in relation to cancer, because it is the main place where the disease is diagnosed, or at least suspected[27]. Previous studies have analysed the association between primary care in health and breast cancer in the United States[28], Saudi Arabia[29], South Africa [30] and in Jordan [31], but no one analysed Brazil.

The results found in these studies performed in other countries were similar from those found in Brazil. Most people in Brazil are attended by the public health system and approximately 75% of the population only has access to health care when made available by the government[32].

However, the socioeconomic barriers and the large territorial extension make Brazil a unique country in a scenario where the development is of a first world country, but the inequality causes it to suffer the triple burden of chronic diseases present in developing countries and with the challenges to managing public health through these inequalities [20].

The more the system is able to bring the population closer to the health service and to provide conditions for cases to be diagnosed, the higher the admission rates will be. The actions carried out in primary health care have the following main objectives: to educate the population in health as a way of promoting health, to increase access to services for diagnosis and treatment, and to perform actions to follow-up patients treated[32].

In this study, we found that increase of PHC coverage in Brazilian municipalities was related to higher rates of the breast cancer mortality. This seems to occurs because the diagnosis does of the breast cancer depend on the actions of health professionals and must, therefore, be based on a triad that also involves the population and the health service [3].

Primary care professionals should initially be able to confirm suspicions or screen individuals with signs and/or symptoms present. The service must support specificity in diagnosis and consequent referral for treatment in a timely manner after diagnosis [33].

One aspect that draws attention to the results is that their relation between breast cancer mortality and Community Health Agents and Family Health Team coverage. Family health strategy aims to diagnose cases when they are less severe, and act in primary care, primarily using strategies to raise public awareness about the signs and symptoms of breast cancer and increasing sensitivity in screening new cases, especially for gaining access to communities that health promotion strategies fail to achieve[34].

Health professionals working in primary care also have the function of carrying out educational activities for health promotion. Among these professionals, nurses are the first to have contact with the population and, consequently, to carry out educational actions [35].

A recent integrative review by Ohl et al. [36] showed that the knowledge of professionals working in primary care in Brazil negatively influence important aspects related to breast cancer, such as breast self-examination, clinical breast examination, mammography examination and factors related to breast cancer screening.

However, this is not exclusive to Brazil. In a study carried out in Nigeria on the knowledge of nurses working in primary care on breast cancer, Oluwatosin [37] reported that despite demonstrating knowledge, nurses working in primary care do not take actions to disseminate knowledge about breast cancer to the population.

In this study, proportion of proportion of people with years-old having completed elementary education and Gini index were related to decrease of mortality was related to the decreasing of the breast cancer mortality while CHAs was related to increase. CHAs are individuals who reside in communities and gain greater access to the most vulnerable populations, rural populations and those with the lowest socioeconomic conditions. CHAs are the members of the team that make the most regular visits to the population, mainly to low-educated populations, prioritising vulnerable populations and access to the health service[24], mainly in places with high-income inequality and by impact of the income inequality to breast cancer in Brazil [5,6,38].

The training of these professionals directly inserted in the communities can be a vector in the dissemination of knowledge about the signs and symptoms that can reach areas where conventional prevention programs cannot, according to the socioeconomic level and education of the population.

For example, a family in poverty living in a rural area will not be exposed to breast cancer prevention campaigns when compared to a high-income family in an urban area, which is not in line with the principles of equity of the Single Health of Brazil, and "Health as a right of all" as ensured in the Brazilian Constitution of 1988.

## Conclusion

The increase in primary care coverage was related to increase the breast cancer mortality in Brazil. In this sense, strategies to improve the quality of primary care professionals to raise awareness about breast cancer, reduce of the income inequality and improve elementary scholarly should be taken into account in the development of public policies in the Brazilian municipalities.
